# EasyDAM_V4: Guided-GAN-based cross-species data labeling for fruit detection with significant shape difference

**DOI:** 10.1093/hr/uhae007

**Published:** 2024-01-10

**Authors:** Wenli Zhang, Yuxin Liu, Chenhuizi Wang, Chao Zheng, Guoqiang Cui, Wei Guo

**Affiliations:** Information Department, Beijing University of Technology, Beijing 100022, China; Information Department, Beijing University of Technology, Beijing 100022, China; Information Department, Beijing University of Technology, Beijing 100022, China; Information Department, Beijing University of Technology, Beijing 100022, China; Information Department, Beijing University of Technology, Beijing 100022, China; Graduate School of Agricultural and Life Sciences, The University of Tokyo, Tokyo 188-0002, Japan

## Abstract

Traditional agriculture is gradually being combined with artificial intelligence technology. High-performance fruit detection technology is an important basic technology in the practical application of modern smart orchards and has great application value. At this stage, fruit detection models need to rely on a large number of labeled datasets to support the training and learning of detection models, resulting in higher manual labeling costs. Our previous work uses a generative adversarial network to translate the source domain to the target fruit images. Thus, automatic labeling is performed on the actual dataset in the target domain. However, the method still does not achieve satisfactory results for translating fruits with significant shape variance. Therefore, this study proposes an improved fruit automatic labeling method, EasyDAM_V4, which introduces the Across-CycleGAN fruit translation model to achieve spanning translation between phenotypic features such as fruit shape, texture, and color to reduce domain differences effectively. We validated the proposed method using pear fruit as the source domain and three fruits with large phenotypic differences, namely pitaya, eggplant, and cucumber, as the target domain. The results show that the EasyDAM_V4 method achieves substantial cross-fruit shape translation, and the average accuracy of labeling reached 87.8, 87.0, and 80.7% for the three types of target domain datasets, respectively. Therefore, this research method can improve the applicability of the automatic labeling process even if significant shape variance exists between the source and target domain.

## Introduction

With the combination of traditional agriculture and artificial intelligence technology, the construction of smart orchards has received more extensive attention in the development of the fruit industry. High-precision fruit detection technology plays a pivotal role in modernized orchards, offering versatile applications including fruit localization, sorting, yield prediction, and automated harvesting. Moreover, it serves as a potent means to gather phenotypic data encompassing texture, size, color, and shape attributes from fruit. These data are integral to advancing plant phenomics research, enabling precise characterization of fruit phenotypic traits. Large-scale fruit data analysis facilitates the accurate measurement and evaluation of these characteristics. Notably, researchers [1] have demonstrated that analyzing physiological traits, such as fruit shape, unveils patterns of variation linked to diverse genotypes and environmental conditions, shedding light on plant evolutionary pathways and determinants. Furthermore, by examining physiological attributes like fruit color in various settings, researchers [2] can forecast the influence of fruit peel color on nutritional components, thereby guiding plant breeding and selection strategies. Consequently, acquiring phenotype information via fruit detection holds substantial reference value for crop genetics, variety enhancement, agricultural production, and food safety [3–5]. Currently, most fruit detection technologies use deep learning methods, which need to rely on a large number of labeled datasets to support the training of deep learning models. Moreover, the fruit trees in real scenes are densely distributed, the fruit growth is irregular, the scale is small, and the shading is serious, leading to a strong scene environment diversity. Due to the poor generalization performance of deep learning models at this stage, researchers need to produce new fruit datasets for different scene environments and different kinds of fruit. This leads to much more difficult and time-consuming labeling of datasets. Furthermore, acquiring and analyzing fruit phenotypic data often entails many labor-intensive experimental and measurement processes, consequently elongating the analysis cycle of fruit phenotypic parameters. This extended timeframe increases the demand for human resources and impedes the pace of advancements in fruit research and studies [6]. Therefore, there is a very urgent need to establish an automatic labeling technique for fruits with high generalization and strong domain adaptation. Hence, an immediate imperative exists to develop an automatic fruit labeling technique characterized by robust generalization and domain adaptation capabilities. The introduction of automatic annotation promises to allow extensive fruit datasets to be expeditiously amassed, a cornerstone for facilitating the high-throughput and precise extraction of plant phenotypic parameters. Consequently, this breakthrough propels advancements in biological research and the practical applications of plant phenomics within the agricultural domain. Such progress is poised to play a pivotal role in realizing high-precision orchard management and crop monitoring, thus providing indispensable support for the sustainable evolution of agricultural production.

The EasyDAM family of methods was proposed in previous research work. Among these methods, EasyDAM_V1 [7] focuses on the automatic labeling of fruit datasets with similar shapes, and EasyDAM_V2 [8] improves the generative adversarial network (GAN) model. Translation of fruit images with small shape differences can be performed, e.g. the translation of round to oval fruit images. By solving the miniature deformation problem, the applicability of the automatic fruit labeling task is improved to some extent. However, the method still cannot solve the large deformation problem. Since the random selection of source domain fruit in EasyDAM_V1 [7] and EasyDAM_V2 [8] has the problem of unstable image generation quality of target domain fruit due to the selection difference, EasyDAM_V3 [9] proposes an optimal source domain establishment method based on a multidimensional spatial feature model to select the most suitable source domain, which can correspond to the multiple types of target domain fruit by one type of source domain fruit, making the model’s generalization greater. The pear is selected as the optimal source domain for experimental verification. Meanwhile, by constructing a knowledge graph of synthesis rules for orchard scene-level components, a large-volume dataset construction method is proposed, which is able to obtain fruit labeling information automatically, providing a foundation and new ideas for subsequent study. To further improve the translation performance of the EasyDAM model in fruit targets with large feature differences (e.g. pear to cucumber), we investigate image translation models for accurate generation of shape, color, and texture features. It is hoped that more realistic multi-category target domain fruit images can be generated from single-category source domain fruit images. This will further improve the automatic generation of labels for target domain datasets with large differences in phenotypic features.

The color features between different domains were realistically transformed in our previous study. Shape and texture features, as two other important basic features describing the fruit phenotype, are essential for generative adversarial network model training to learn realistic fruit features [10]. They can help target detection models to extract foreground target features better. Few researchers in agriculture have focused on the shape, texture, and color features of fruits to control the generation of fruit images in the target domain. In agricultural orchards, there are many kinds of fruits, and the fruit features of different kinds or varieties vary greatly. It is still difficult to further reduce the domain differences and achieve realistic translation of fruits across the domain. Therefore, in this paper, based on a detailed technical survey for the study of generative adversarial networks in image translation tasks such as shape and texture, a novel technique and idea for spanning translation between fruit phenotypic features is proposed.

For **shape features**, some researchers have introduced supervised signals for shape features to train generative adversarial network models better. Mo *et al*. [11] proposed an InstaGAN based on instance information. The model extracts the color and shape features of the target by jointly encoding the RGB image of the target and the instance mask image. Chen *et al*. [12] proposed a conditional variational generative adversarial network (CVGAN), which solves the problem of unsatisfactory image transformation when the shape difference between different domains is large. By modeling the intrinsic interactions between shape and appearance, the desired image is generated and guided by the target shape. The model allows human intervention in shape variation, thus generating different images of the person. Roy *et al*. [13] proposed a semantics-aware generative adversarial network model. This model learns foreground object features by using the instance mask information of the target as a supervised signal. A cross-domain semantic consistency loss function is further proposed to preserve the geometric structure semantic information to achieve shape transformation between targets. Chen *et al*. [14] proposed a deep generative network, DECOR-GAN, for 3D shapes. By inputting the style encoding of samples as conditional information into the model, a realistic transformation of the detailed part of the 3D model is achieved. However, the supervised training method can achieve a better shape change effect but requires a large amount of label data. It is more time-consuming and laborious in practical applications, and achieving good results in practical landing tasks is difficult. Therefore, some researchers have done a lot of research work in the field of unsupervised image translation [15]. Therefore, some researchers have done much research in unsupervised image translation. Wu *et al*. [15] proposed a framework for image translation tasks by introducing geometric loss and conditional variational autoencoder (VAE) loss for representing the shape features of the target. Li *et al*. [16] proposed SP-GAN, which guides the whole model generation process by introducing global prior knowledge and establishing point-to-point correspondence for multi-shape component generation. Zhang and Hou [17] introduced an adversarial consistent loss function in generative adversarial networks and used it together with other loss functions to guide model training. The semantic information in the original image is effectively preserved for shape translation of the target. Gokaslan *et al*. [18] introduced a discriminator with extended convolution. It can use global image information to train a more context-aware generator for the shape-changing image translation task. Huang *et al*. [19] proposed the SoloGAN model. By sharing encoders and generators between all domains, domain invariant features are extracted efficiently. Some results have been achieved in the task of shape-changing image transformation. Our previous research work [8] compared the existing shape translation methods and proposed a method to calculate the structural consistency loss based on the cross-loop structure. By adding a multiscale fusion module this can realistically translate to get the target domain images with certain shape differences. The domain differences of fruits between different domains are reduced. Although the existing methods can achieve certain shape changes with sufficient dataset samples, it is more difficult to extract effective feature information in complex tasks due to the unsupervised methods’ own lack of accurate supervised signals. It is also impossible to realize the conversion between targets with large shape changes.

For **texture features** some researchers have increased the resolution of the generative adversarial network model, resulting in or more detailed texture features. Hedjazi and Genc [20] designed a progressive generative adversarial network model. The detailed features of the generated images are controlled by four generators and discriminators with different resolutions to achieve finer-grained transformations. Hu [21] proposed Multi-Texture GAN. They designed a multiscale texture transfer algorithm. By computing the texture similarity between different images, it is recovered into the target image as *a priori* knowledge using a matching mechanism. Thus, the detailed features in the reconstructed images are enriched. At this stage, more and more researchers choose to use potential space to accurately generate texture features. Karras *et al*. [22–24] proposed the StyleGAN family of models. Different detail features in the image are separated by designing latent encoding and generating random variations for different style texture features. Thus, images with more realistic and detailed texture features are generated. Wu *et al*. [25] proposed the cycle consistency assumption and the SDSGAN model with a shared deep space. The two images are encoded into a shared deep space by a pre-trained VGG19 network. The texture features are then translated into the corresponding image domains using two decoders. Johnson *et al*. [26] extracted high-level features through a pre-trained backbone network and proposed to train the feedforward network by calculating the loss between prediction and ground truth using a perceptual loss function. While improving in speed, some results were achieved in texture detail translation. Liu *et al*. [27] proposed a shared potential space hypothesis. It is proposed that the joint distribution between two different domains can be used to derive the respective marginal distributions, thus further reducing the domain differences in the image translation task. Good results were achieved in the high-resolution image translation task. Bergmann *et al*. [28] proposed the Periodic Spatial GAN (PSGAN) model. A smooth interpolation operation is performed from the structured noise space to sense the texture sample information of the images in the original dataset. Thus, the periodic samples in the target are learned precisely. The method is currently more widely used in face image translation tasks. Shen *et al*. [29] proposed an InterFaceGAN framework to interpret the information of the unentangled face representations learned by existing GAN models and studied the nature of face semantics encoded in the potential space. Thus, realistic translation of face images under different poses is achieved. Sainburg *et al*. [30] proposed an autoencoder (AE) and GAN-based generative network structure. The convex potential distribution is facilitated by adversarial training on potential space interpolation. Thus, different attributes in the target are controlled to reach a more detailed variation in the face image. However, the above techniques consider more detailed features of the sample target. Thus, the generated samples are more detailed. In the case of agricultural orchards, more attention needs to be paid to the texture of the fruit and the correlation of the structural arrangement of the surface. Therefore, it is not common in the field of agriculture.

In summary, the existing research techniques focus more on the direction of generating image diversity and the control of single features. Most of the research results demonstrate the modification of the network structure from a macroscopic perspective. However, for unsupervised learning tasks such as GAN models, there is still a need to enhance and improve how to effectively extract multi-dimensional features from the desired foreground targets and control the accurate generation of multi-dimensional features to reduce the domain discrepancy problem further. Therefore, this paper proposes an improved fruit automatic labeling method, EasyDAM_V4, based on the previous studies’ EasyDAM_V1, EasyDAM_V2, and EasyDAM_V3. It is mainly based on the miniature morphological fruit translation model Across-CycleGAN from the previous study. A GAN model, Guided-GAN, is designed to achieve spanning translation among phenotypic features such as fruit shape, texture, and color, which can more accurately control the learning of multi-dimensional phenotypic feature parameters by the generator. The more realistic translation is performed for fruits with large phenotypic differences in the target domain, effectively reducing domain differences. This enables the automatic labeling of high-quality multi-category fruit datasets. The algorithm contains the following main innovations:

(i) There is significant variation in the shape, color, and texture features of different fruits. Unsupervised learning usually makes it more difficult to extract features from a large amount of unlabeled data. However, it is essential to find the most significant phenotypic features of fruits in spanning image transformation tasks with significant feature differences [31, 32]. Therefore, a multi-dimensional phenotypic feature extraction method based on potential space is proposed in this paper. The solution space representation of salient features in the latent space is utilized. The multi-dimensional feature maps of fruits are effectively extracted, and they are input into the model together with the original RGB images for training, which enables the GAN to better extract the different phenotypic features of the fruit targets.

(ii) The existing image translation models are not accurate enough for the description of fruitful features in the translation of targets with large feature differences due to the problem of functional singularity of the loss function, which leads to the loss function not converging to a suitable interval during the training process. Thus, the generation direction of the model in the multi-dimensional features cannot be well controlled. Therefore, this paper proposes a multi-dimensional loss function design method based on the entropy weight method. By designing and adding three different loss functions, they are used to accurately describe the multi-dimensional phenotypic features of fruits in the training process. The weights of the loss functions are dynamically controlled by the entropy weight method so that the generation direction of the fruit images in the target domain can still be controlled in the spanning fruit translation with large differences in features, especially in shape features.

## Materials and methods

**Figure 1:**
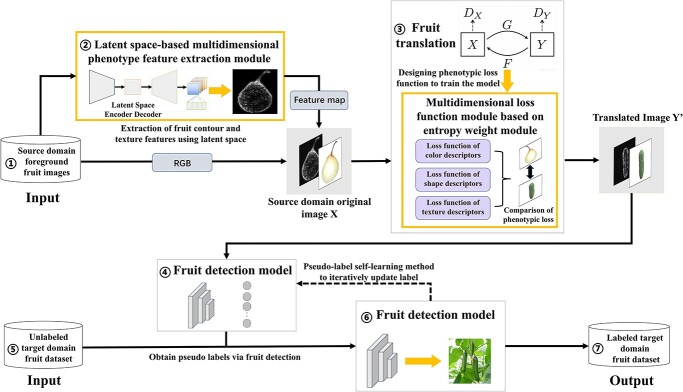
Overall architecture of the EasyDAM_V4 method. The overall architecture of the proposed method involves seven main parts. The source domain foreground fruit image represented by (1) and the labeled target domain fruit dataset represented by (7) are the input and output of the method, respectively. Boxes (2) and (3) illustrate the main innovation points of this paper. (2) The shape texture feature map obtained by using the latent space-based multi-dimensional phenotype feature extraction method. The fruit translation model is trained together with the original RGB image after concatenation. (3) The multi-dimensional loss function module based on the entropy weight method, which is used to accurately describe the phenotypic features of fruits during the training process. Rectangles of different colors indicate the fruit image translation results controlled by different categories of loss functions.

In this paper, based on the previous studies of EasyDAM_V1, EasyDAM_V2 and EasyDAM_V3, we mainly design a GAN model, Guided-GAN, for fruit target translation from the source domain to the target domain with large feature differences. The overall architecture of automatic labeling using the proposed method is shown in Fig. 1, where the yellow boxes show the main innovations of this paper. The details of each innovation point are described as follows.

(i) In order to effectively extract essential phenotypic features in different classes of fruit targets and improve the unsupervised model in which it is more difficult to learn significant feature information of targets, a multi-dimensional phenotypic feature extraction method based on latent space is proposed in this paper (see the Dataset introduction section for details). This is shown in box 2 in Fig. 1. The method extracts fruit phenotypic features using latent space and feeds the fruit images into a pre-trained VGG16 backbone network. The set of shape as well as texture feature vector solutions (i.e. solution space representation) is highlighted. The guided back-propagation method [33] is used to visualize the shape as well as texture information in the fruit target to form a grayscale feature map. It is fused with the original RGB image to achieve improvement of the input image signal of the network model. It is also fed into the GAN as multimodal solution space data to assist the network model to better learn the phenotypic features of the fruit.

(ii) In addition, to address the problem that the EasyDAM_V2 method in the previous study chose to use cycle-consistent loss to describe the color as well as texture features of the fruit simultaneously, resulting in less accurate feature description, this paper proposes a multi-dimensional loss function design method based on the entropy weight method (see the Experimental strategies section for details), as shown in box 3 in Fig. 1. One or more loss functions are used to describe the phenotypic features of a single category. The size of the difference between the single category features of different fruits is calculated based on the quantifiability description results of the phenotypic features of different categories of fruits. Finally, the weight relationship between different loss functions is dynamically adjusted according to the difference value using the entropy weight method. For the fruit texture features, a texture loss function based on differentiable local binary pattern (LBP) descriptors is introduced in this paper. The fruit shape features follow the cross-loop multiscale structural similarity loss function describing the shape features that performed well in the previous study [8]. The color features are described jointly using cycle-consistent loss as well as identity loss.

In our previous study [8], the source domain fruit images were translated into simulated target domain fruit images by GANs and were constructed as a synthetic dataset. Then, we input to the anchor-free detector-based fruit detection model for feature extraction and training learning of fruit targets to obtain pseudo-labels of the target domain dataset. The label information is updated in each cycle by using the pseudo-label self-learning method with adaptive threshold selection strategy. Finally, the fruit label information of the actual scene fruit dataset in the target domain is obtained to achieve the purpose of automatic labeling.

### Multi-dimensional phenotype feature extraction method based on latent space (box 2 in Fig. 1)

At this stage, it is difficult to extract essential semantic features from the network effectively using unsupervised learning due to the use of unlabeled data for training and learning, which leads to poor target feature presentation capability of unsupervised learning methods. As latent space techniques show strong potential in more and more domains [34–36], applying them to generative networks to extract essential features of targets in different domains can further improve network performance. Thus, some more complex tasks can be achieved. However, in the field of agricultural fruits it is more difficult to extract features of the surface arrangement structure of fruits. Therefore, we need to focus more on the multi-dimensional phenotypic features of fruits. Decomposing the features of fruit targets into multiple interpretable attributes through latent space is used to better extract shape and texture features.

**Figure 2:**
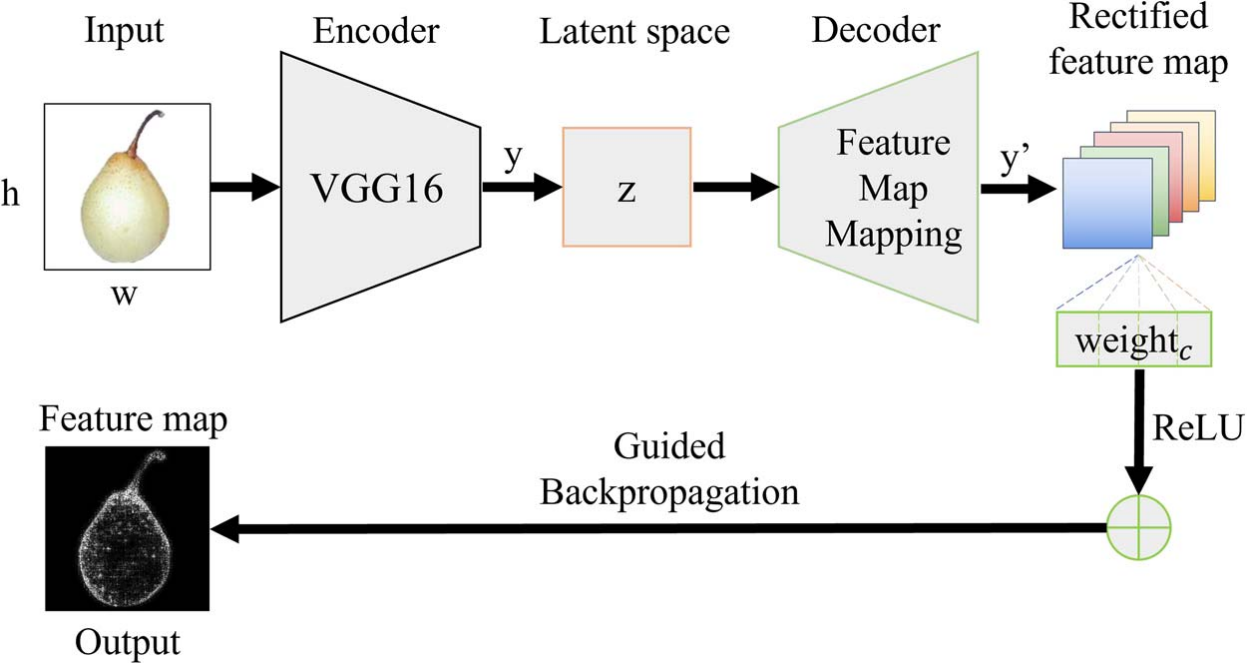
Flow chart of the multi-dimensional phenotype feature extraction method based on latent space.

To this end, as shown in Fig. 2, this paper utilizes a pre-trained backbone network as an encoder to explore the latent space of fruit images. Backward-guided feature visualization mapping is utilized as a decoder to highlight the solution space representation of fruit features. The implementation uses an unsupervised approach to discover potential features in the fruit target and fuses them with the original RGB image to form a multimodal input signal that is jointly input to the fruit translation network. For the selection of the backbone network, considering that the encoder should correspond to the decoder construction, this paper chooses to use the serialization network VGG16 as the encoder. In order to better decouple the image shape texture semantic features, this paper chooses to extract its high-level semantic information from the vectorized representation of the output image of the deep convolutional layer of VGG16 in the last layer. This vector value, *y*, is feature-decoupled using the latent encoding *z* and the feature map mapping is performed by the decoder. The gradient information *y*′ of each feature in the deep convolutional layer is obtained, which can be expressed as the contribution of each channel in the convolutional layer to *y*. The larger the contribution, the more important the channel is considered by the network. The contribution value of each channel is denoted as ${weight}_c$. Back-propagation is then performed. The activation gradient of the image is calculated by the ReLU activation function and weighted summation. The advantage of this is that no adjustment of the input image is required and the deep and complex feature information can be learned effectively. We also guide the back-propagation process to limit the back-propagation of gradients less than 0. The importance of each channel is obtained by normalizing *y*′ to the mean of the width and height in the feature map. Doing so maximizes the high-level semantic feature images in the activation target. Finally, the shape texture feature map of each type of fruit image can be obtained after spatial decoupling. The computational process of obtaining the shape texture feature maps can be expressed as the following equations:

(1) \begin{equation*} {weight}_c=\frac{1}{w\ast h}\sum_i\sum_j\frac{\partial y}{\partial{(Conv)}_c^{ij}} \end{equation*}

(2) \begin{equation*} FeatureM\mathrm{a}p= ReLU\left(\sum_c{weight}_c\ast C{onv}_c\right) \end{equation*}

where ${weight}_c$ denotes the weight share for the $c$ channels in the feature layer $Conv$; $y$ denotes the vector value obtained from the original image after forward propagation of VGG16; $w$ and $h$ denote the width and height of the feature image, respectively; and ${(Conv)}_c^{ij}$ denotes the data at coordinate position $\left(i,j\right)$ in channel $c$ for the feature layer.

### Multi-dimensional loss function design method based on entropy weight method (box 3 in Fig. 1)

In order to more accurately describe the phenotypic features of fruits with large feature differences and to solve the problem of functional homogeneity of loss functions, this paper proposes a multi-dimensional loss function design method based on the entropy weight method. The generation direction of multi-dimensional features is better controlled in the fruit image translation model. Finally, it achieves a better result even in the spanning fruit translation task with large feature differences; see the section Multi-dimensional loss function design and comparison method (in Guided-GAN), and the section Dynamic adaptive weighting method based on the entropy weighting method.

#### Multi-dimensional loss function design and comparison method

To address the problem that GANs have difficulty in accurately describing features such as shape and texture, some researchers [37, 38] proposed to introduce instance-level loss constraints to better regulate the generation direction of foreground targets in images. However, this approach is not applicable to unsupervised learning-based approaches due to the introduction of a manual labeling process. It is not suitable for the unsupervised learning-based fruit automatic labeling task. In contrast, our team improved a fruit conversion model, Across-CycleGAN, based on the CycleGAN image translation algorithm in a previous study [8]. By introducing a structural similarity loss function, we achieved the conversion from circular to elliptical fruits. In order to better improve the generalization of the automatic fruit labeling method, methods need to be designed to precisely control the model’s source-domain to target-domain fruit generation direction in the spanning fruit image conversion task with large differences in phenotypic features. In order to further improve the performance of the unsupervised fruit translation model. Thus, more kinds of target domain fruits can be automatically labeled for a wider range of requirements.

**Figure 3:**
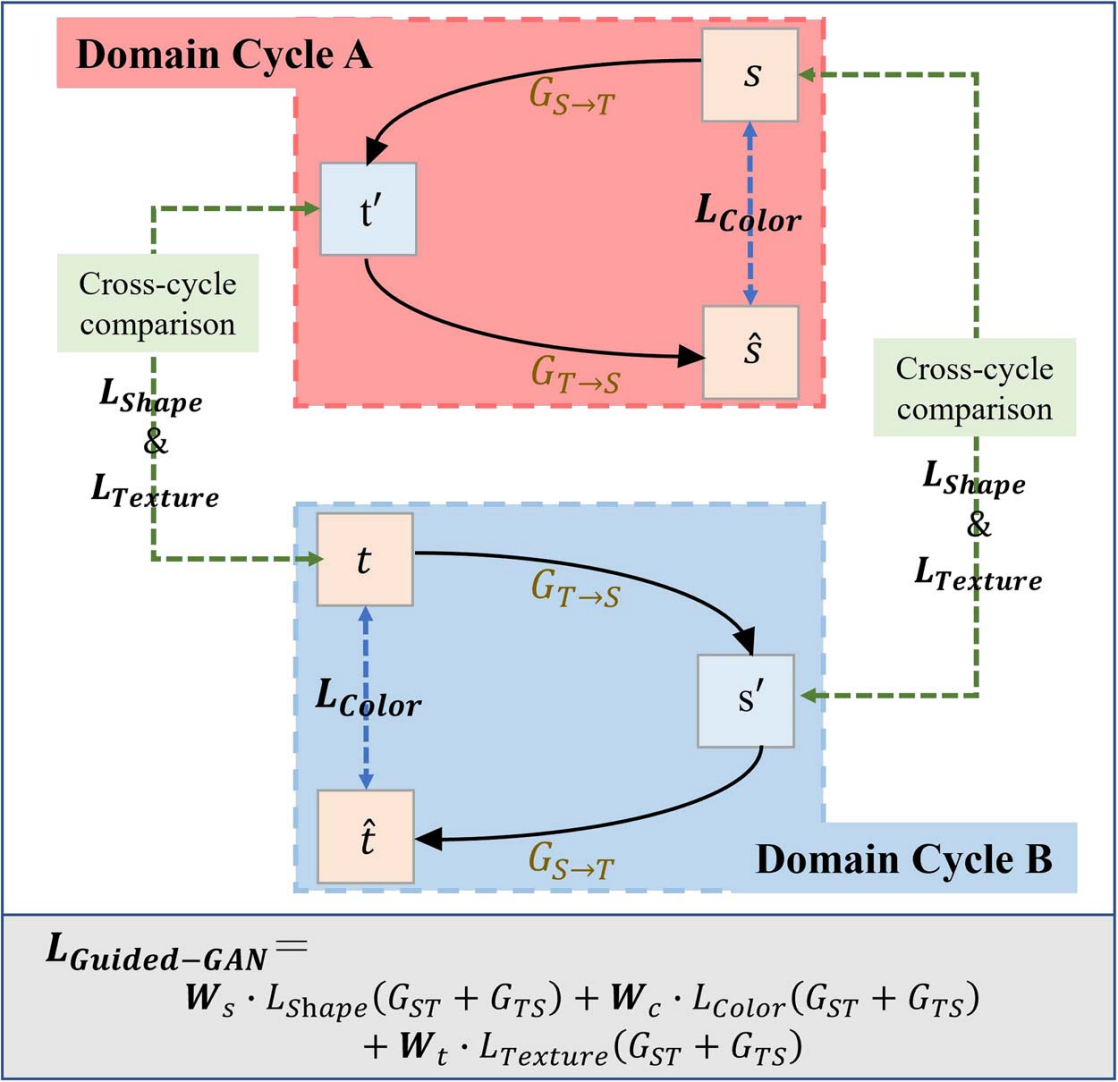
schematic diagram of multi-dimensional loss function in the Guided-GAN model. The model contains two generators, ${G}_{S\to T}$ and ${G}_{T\to S}$. There are three types of loss functions involved, namely ${L}_{Color}$, ${L}_{Shape}$, and ${L}_{Texture}$.The domain cycle is divided into two parts, Domain Cycle A and Domain Cycle B, which are used to control the generation of color features of fruits. The cross-cycle comparison text box indicates the cross-cycle loss function comparison path. In the training process of different domain cycles of the network, the real fruit image feature information is used to train the fitting network to generate simulated fruit image data. It helps the model to learn better and constrain the generation of shape and texture features.

Based on this, this method is designed to use multi-dimensional loss functions to constrain the color, shape, and texture generation direction of fruits during the training of the fruit translation model. The schematic design of the multi-dimensional loss function in the generator of the model is shown in Fig. 3. In this paper, two generators and two discriminators are used to construct two cycle training structures, Domain Cycle A and Domain Cycle B, respectively. Two loss function comparison schemes are combined with cross-cycle training (e.g. the ${L}_{Shape}\&{L}_{Texture}$ parts on both sides in Fig. 3) and within-cycle training (e.g. the ${L}_{Color}$ part in the middle of Fig. 3) to accurately describe the color, shape, and texture features, respectively.

The overall loss function ${L}_{\mathrm{G} uided- GAN}$ of the generator part of this model can be expressed as:

(3) \begin{align*} {L}_{\mathrm{G} uided- GAN}&= {\boldsymbol{W}}_s\bullet{L}_{S\mathrm{h} ape}\left({G}_{ST}+{G}_{TS}\right)+{\boldsymbol{W}}_c\bullet{L}_{Color}\left({G}_{ST}+{G}_{TS}\right)\notag\\ &\quad+{\boldsymbol{W}}_t\bullet{L}_{Texture}\left({G}_{ST}+{G}_{TS}\right)\end{align*}

where ${G}_{ST}$ denotes the generator that maps the source domain to the target domain, ${G}_{TS}$ denotes the generator that maps the target domain to the source domain, and ${\boldsymbol{W}}_s,{\boldsymbol{W}}_c$, and ${\boldsymbol{W}}_t$ denote the proportion of weights assigned to the shape, color, and texture loss functions during model training using the entropy weight method, respectively.

For the **color feature loss function**, in this paper we follow the cycle-consistent loss function and identity loss in the CycleGAN network. Its excellent coloring effect can help the fruit translation model to better control the generation of color features. The cycle-consistent loss can be expressed as:

(4) \begin{equation*} {L}_{Color}\left({G}_{ST}+{G}_{TS}\right)={L}_{Cycle}\left({G}_{ST}+{G}_{TS}\right)+{L}_{Identity}\left({G}_{ST}+{G}_{TS}\right) \end{equation*}

(5) \begin{align*}\notag{L}_{Cycle}\left({G}_{ST}+{G}_{TS}\right)&={E}_{s\sim pdata(s)}{\left\Vert{G}_{TS}\left({G}_{ST}(s)\right)-s\right\Vert}_1\\&\quad +{E}_{t\sim pdata(t)}{\left\Vert{G}_{ST}\left({G}_{TS}(t)\right)-t\right\Vert}_1 \end{align*}

The identity loss function can be expressed as:

(6) \begin{equation*} {\displaystyle \begin{array}{c}{L}_{Identity}\left({G}_{ST}+{G}_{TS}\right)={E}_{s\sim pdata(t)}{\left\Vert s-{G}_{ST}(s)\right\Vert}_1+{E}_{s\sim pdata(t)}{\left\Vert t-{G}_{TS}(t)\right\Vert}_1\end{array}} \end{equation*}

where $s\sim pdata(s)$ and t$\sim pdata(t)$ denote the data distribution in the source domain and the target domain, respectively, and $t$ and $s$ denote the image information in the target domain and the source domain, respectively.

For the **shape feature loss function**, in this paper we choose to use MS-SSIM [39] based on a multiscale structural similarity index. Different size convolution kernels are used to adjust the image perceptual field size and count the shape structure feature information of the corresponding region of the image under different scale conditions. It can effectively distinguish the geometric differences between different classes of fruit images and train the model to better adapt to the variation of shape features between different classes of fruits. In this paper, the original image is compared with the translated image in another cycle by using the cross-loop comparison method. Thus, the process of generating fruit shape features is better constrained. It can be expressed as follows:

(7) \begin{align*} {L}_{Shape}\left({G}_{ST}+{G}_{TS}\right)&=\left(1- MS\_ SSIM\left({G}_{ST}(s),t\right)\right)\notag\\&\quad +\left(1- MS\_ SSIM\left({G}_{TS}(t),s\right)\right) \end{align*}

where $MS\_ SSIM$ denotes the calculation based on multiscale structural similarity index loss.

For the **texture feature loss function**, because the texture features in the fruit images are too detailed, the texture features cannot be fully expressed if the comparison of the loss function is performed only from the original RGB images. The resolution of the fruit in the dataset is much smaller. The texture features are even less well represented. It adds some difficulty to the image translation model. Therefore, this paper designs a texture feature loss function based on the local binary pattern (LBP) descriptor to make it better highlight the texture of the fruit and its regular arrangement of the texture loss calculation method, and accurately describe its texture features, and to provide better performance of the image translation model. It can be expressed as:

(8) \begin{equation*} {\displaystyle \begin{array}{c}{L}_{Texture}\left({G}_{ST}+{G}_{TS}\right)= Pearson\left( LBP\left({G}_{ST}(s),t\right)+ Pearson\left({G}_{TS}(t),s\right)\right)\end{array}} \end{equation*}

where $LBP\left(X,Y\right)=N\left( LBP\left({x}_C,{y}_C\right)\right)$, $LBP\left({x}_c,{y}_c\right)={\sum}_{p=0}^{P-1}{2}^ps\left({i}_p-{i}_c\right)$, and $s(x)=\begin{cases}1\ if\ x\ge 0\\{}0\ else\end{cases}$, $Pearson$ denotes the magnitude of variability among fruit texture features using the Pearson correlation coefficient, $N$ denotes traversal of all pixel values in the whole image, ${x}_C,{y}_C$ denotes the central pixel, $i$ denotes the grayscale value, $s$ is the symbolic function, and $P$ denotes the P-neighborhood selected from the central pixel point. The scale of the input image in this study is 256 × 256, and it is experimentally verified that $P$ is best when it is 16 at this image resolution.

The distribution of the two image domains is highly discrete and irregular in the absence of pairwise supervised information constraints. In this paper, the multi-dimensional loss function is designed to constrain the direction of visual attributes such as color, shape, and texture of fruits during the training of the fruit translation model. The multi-dimensional phenotypic features in the fruit translation process can be described more accurately.

#### Dynamic adaptive weighting method based on the entropy weighting method

In the section Multi-dimensional loss function design and comparison method, we designed the loss function part of the GAN model. Adding a multi-dimensional feature loss function is used to accurately characterize the fruit during the training process. However, when designing a GAN network, it is not the case that the higher the number of loss functions, the better trained the model will be. If too many loss functions are added, the model will not fit properly in the training phase and thus lose the generative direction of describing the fruit features. In order to balance the multi-dimensional loss functions added in the GAN model, it is usually necessary to manually adjust the weights of different loss functions based on empirical values so that they can converge stably. However, the differences in the phenotypic characteristics of different classes of fruits are very obvious. It is difficult to precisely control the fruit generation direction from the source domain to the target domain using the same weights, which makes it more difficult to realize the spanning translation between fruit phenotypic features. In order to balance multiple feature loss functions concisely and effectively, a dynamic adaptive weighting method based on quantifiable fruit phenotypic features is introduced in this paper. It is used to automatically level the weights of multi-dimensional loss functions among multiple categories of fruits. It gets rid of the drawback of not being accurate and efficient enough when setting empirical values to level the weights.

In this paper we directly compare the magnitude of variability of shape, color, and texture descriptors of all samples of the two types of fruits when performing the conversion between the two types of fruits. The specific values of the variability between their fruits are automatically calculated. It also dynamically adjusts the weight ratio of the multi-dimensional loss function at each training session. Thus, it better assists the network model for fitting and accelerates the convergence process. This results in better quality of the generated fruit images in the target domain.

The specific process of the method is as follows. First, the quantifiable descriptor values of the *i*th fruit shape, color, and texture features in the source and target domains are calculated in turn, and normalized. They are denoted as $ {S}_i,{C}_i,\mathrm{and}\ {T}_i $, respectively.

Next, the specific weight ${P}_{ij}$ is calculated for each fruit sample under different eigenvalue labels. It is used to describe the magnitude of variability in the values of different feature descriptors, as shown in Equation (9).

(9) \begin{equation*} {\displaystyle \begin{array}{c}{P}_{ij}={Y}_{ij}/\sum_j{Y}_{ij}\end{array}} \end{equation*}

where j takes the three characteristics of $S,C,\mathrm{and}\ T$ in turn as three different indicators.

Next, according to the definition of information entropy in information theory, the larger the value of the descriptor variance of different fruit samples, the more information can be provided in the training of the GAN model. Therefore, more weights need to be assigned to them during the model training process. At this time, the information entropy of a set of data is calculated as shown in Equation (10).

(10) \begin{equation*} {\displaystyle \begin{array}{c}{E}_j=-\ln{(n)}^{-1}\sum_{i=1}^n{P}_{ij}\ \mathit{\ln}{P}_{ij}\end{array}} \end{equation*}

Finally, the weight of each index is obtained according to the formula of information entropy:

(11) \begin{equation*} {\displaystyle \begin{array}{c}{W}_j=\frac{1-{E}_j}{j-E}\left(j=S,C,T\right)\end{array}} \end{equation*}

## Experiments and results

In the experimental part of this study, we followed the conclusions related to the optimal source domain selection in EasyDAM_V3 from the previous study. The optimal source domain dataset for most fruits, i.e. pear fruit images, was used as the source domain. Three fruits with large differences in phenotypic characteristics, i.e. pitaya, cucumber, and eggplant, were selected as target domains. Phenotypic feature spanning fruit translation and label generation experiments were conducted. The accuracy of label frame generation was calculated. This can not only verify the generalization of the automatic fruit labeling method in this paper, but also verify the feature conversion performance of the proposed fruit translation model in fruits with large differences in phenotypic features.

### Dataset introduction

In our prior research, we conducted experiments involving the automatic labeling of various fruit types. In EasyDAM_V1 [7], we assessed apples and tomatoes, which share a similar shape to the source domain fruit (orange). EasyDAM_V2 [8] involved the evaluation of mangoes and pitayas, exhibiting minor shape differences from the source domain fruit (orange). Recognizing the issue of random fruit type selection from the source domain in the previous studies [7, 8], we introduced EasyDAM_V3 [9], utilizing pears as the optimal source domain while evaluating oranges, apples, and tomatoes. In this study, we extended our investigation to cucumbers, eggplants, and pitayas, which present substantial shape disparities compared with the source domain fruit. These choices pose a more formidable challenge to the algorithm. In this study, two fruit datasets are used: a transparent background fruit dataset and an actual orchard scene dataset in the target domain. The transparent background fruit dataset is used to train the Guided-GAN to translate the source domain fruit images to the target domain images. After obtaining the generated target domain transparent background fruit images, the synthetic dataset of the target domain is constructed using the synthetic dataset construction method with excellent performance in the previous study [8] and the pseudo-label self-learning method with adaptive threshold selection, and input to the fruit detection model for training as well as iterative update. The label information of the target domain fruits is obtained in the actual orchard scene dataset of the target domain.

#### Transparent background fruit dataset

In order to enhance the domain adaptation of the automatic labeling method in different target domain scenarios and different classes of fruits, we choose to use the method of transparent background fruit image translation and synthetic dataset construction in the previous study. The source domain fruit we choose is pear, and the target domain fruits are pitaya, eggplant, and cucumber. Compared with other commercially available fruit species, the phenotypic features of these three types of fruit are more different and discrete in distribution. Source domain fruit images with similar feature distances could not be selected. The selection of dataset is very suitable for the validation of the proposed method ，and can also provide a new solution to the scenario where the best source domain can never be selected for the image translation task. The resolution in our dataset is uniformly 256 × 256 to allow feature analysis and deep learning model training. The images in the dataset are obtained from the public dataset Fruits-360 [40], actual fruit collections, and internet searches (no copyright restrictions). The fruit images of pear, pitaya, eggplant, and cucumber are shown in Supplementary Data Fig. S8.

#### Actual orchard fruit dataset

To verify the effectiveness of the proposed method in this paper, we collected and photographed the datasets of the actual scenes of fruits in the three target domains mentioned above, as shown in Supplementary Data Fig. S9. In order to ensure the diversity of image samples collected in actual orchards, this study utilized the actual scene shooting combined with internet images. There are certain differences in shooting scenes, shooting environments, shooting distances and shooting angles. These images were captured in the orchard field, with different shooting distances and angles, and under different lighting conditions, such as the existence of near-field, far-field, backlight, etc., which made the features, scales, and occlusion of different targets in the images vary greatly and produced problems such as shape distortion and loss of details. Moreover, most of the cucumber image targets are extremely similar to the background color characteristics. All of the above factors pose significant challenges for this study. The datasets used in this paper have a wide range of applications and richness, and their evaluation can demonstrate the generalization capability of the proposed algorithm in this study.

The details of the three different fruit datasets are shown in Table 1. For each dataset the details are presented as follows:

**Table 1:** Collection information on three types of target domain fruit datasets.

**Type of dataset**	**Collection locations**	**Number of unlabeled images**	**Number of labeled images**
Pitaya	A picking garden in Fangshan District, Beijing, China (latitude and longitude 115.968, 39.615)	265	112
Eggplant	Fruit-262 public dataset [41]	380	136
Cucumber	A picking garden in Fangshan District, Beijing, China (latitude and longitude 115.968, 39.615)	297	83

(i) Pitaya dataset. The images of this dataset were collected from a picking garden in Fangshan District, Beijing, China. The shooting method was handheld using a Samsung GalaxyS8 cell phone (Samsung Electronics Technology, South Korea). To ensure data diversity, we also collected pitaya data from other scenes on the internet, including images from different scenes such as distant, close, indoor, and outdoor. The dataset contains 377 pitaya images. Among them, 265 unlabeled images were used as the training set for the pseudo-labeling self-learning method, and 112 labeled images were used as the test set to verify the effectiveness of the proposed automatic labeling method.

(ii) Eggplant dataset. The dataset images were mainly obtained from the Fruit-262 public dataset [41] and eggplant images were downloaded through an internet search. To ensure the scale size of the target and the diversity of aspect ratios, this dataset contains 516 eggplant images of different varieties from different angles. Among them, 380 unlabeled images were used as the training set for the pseudo-label self-learning method, and 136 labeled images were used as the test set.

(iii) Cucumber dataset. In this paper short-stalked Eurasian cucumbers, which are commonly available in the market, were used as the experimental samples. The images were collected from a picking garden in Fangshan District, Beijing, China, and from the internet. There are 380 images of cucumbers with white spines and without spines. Among them, 297 unlabeled images were used as the training set for the pseudo-labeling self-learning method, and 83 labeled images were used as the test set.

### Experimental strategies

In this study two parts of experimental training were conducted for fruit image translation and fruit image detection, respectively. For the fruit image translation part, the concept of optimal source domain in the fruit auto-labeling task was demonstrated in our previous study. This led to a decrease in the difficulty of image collection and an increase in the fidelity of model training. However, for some fruits with large differences in phenotypic features, the best source domain fruits can never be selected, so the Guided-GAN fruit translation network proposed in this study can be used to achieve the leapfrog translation between fruits with large differences in phenotypic features. To verify the performance of the model proposed in this study, we selected pear as the source domain and pitaya, eggplant, and cucumber as the target domain for fruit image translation. For the fruit image detection part, we complete the automatic fruit labeling by the target detection algorithm CenterNet [42], and finally output the labeled frame information of the fruit images in the target domain. In the training process, we use the synthetic dataset construction method in the previous study to construct a large synthetic dataset of the target domain by establishing the knowledge graph of the component synthesis rules to make a detection model with better domain adaptation, and also use the unlabeled target domain fruit images in the above dataset to iteratively update the detection model by the pseudo-label self-learning method of adaptive threshold selection to filter out low-quality imprecisely labeled frames to further extract the target fruit features and enhance the generalization of the method. No manually labeled label information is used in the overall training process to achieve the automatic fruit labeling task with zero labor cost.

This experiment deployed a deep learning framework for model training and testing on a computer platform with an Intel Core i7-10700K CPU processor (64 GB of RAM), a GeForce RTX 3090 GPU graphics card (24GB of video memory), and an operating system with ubuntu 18.04 LTS, using the Python 3.6.5 programming language to implement the construction, training, and validation of network models under the Pytorch 1.0.0 deep learning framework.

For Guided-GAN model training, the network was trained using a mini-batch adaptive moment estimation (Adam) optimizer with a momentum factor of 0.5 and a batch size of 1. The learning rate for the first 100 training epochs was set to 0.0002, and the learning rate for the next 100 training epochs was set to zero with linear recession.

For CenterNet model training, batch size was set to 4 and the initial learning rate to 0.000125 for 100 training epochs and the learning rate was decreased by a factor of 10 at the 90th training epoch.

### Evaluation metrics

The ultimate goal of the proposed method in this paper is to generate accurate label information for the target domain fruit dataset. Therefore, we input the target domain fruit images obtained by Guided-GAN translation into the detection model to obtain label information. The accuracy of the label frames obtained by the detection model was used as an evaluation index to measure whether the label frames obtained by the method were accurate enough. Thus, the performance of the proposed model in this paper was verified. There were three main metrics used. They were precision (*P*), recall (*R*), *F*1 value, and average precision (AP). Higher values of the corresponding metric indicated higher quality of tag generation in the target domain scenario. In addition, the precision, recall, and *F*1 values used in this paper were taken from the values at the equilibrium point. The *P*, *R*, and *F*1 values were calculated using Equations (12)–(14), respectively:

(12) \begin{equation*} {\displaystyle \begin{array}{c}P=\frac{TP}{TP+ FP}\end{array}} \end{equation*}

(13) \begin{equation*} {\displaystyle \begin{array}{c}R=\frac{TP}{GT}\ \end{array}} \end{equation*}

(14) \begin{equation*} {\displaystyle \begin{array}{c}F1=\frac{2\ast P\ast R}{P+R}\ \end{array}} \end{equation*}

where TP indicates the number of true-positive samples. In the target domain, the fruit dataset indicates the number of fruits with correct automatic labeling results. FP indicates the number of false-positive samples. In the target domain, the fruit dataset indicates the number of samples in which the non-fruitful targets are incorrectly labeled. GT indicates the number of fruitful samples in the test set obtained by the manual labeling method. The formula shows that we expect a high TP value as well as a low FP value. The average accuracy AP is used as a standard measure to evaluate the sensitivity of the network in detecting fruit targets with respect to the target object. It is used in this experiment to synthetically represent the performance of the automatic labeling method. Its physical meaning is the area of the *P*–*R* curve, which is the average of the precision *P* as the recall *R* varies in the interval 0–1. It can be calculated using Equation (15):

(15) \begin{equation*} {\displaystyle \begin{array}{c} AP={\int}_0^1{P}_{(R)} dR\end{array}} \end{equation*}

where ${P}_{(R)}$ represents the precision *P* as a function of recall *R* at different confidence levels. Since the performance comparison results are not the same for different confidence levels, the performance of different networks will be determined mainly by the value of AP in this paper. Meanwhile, the rest of the evaluation values will be given in the paper for further study and analysis.

### Results

In the experimental part of this study, we implemented unsupervised automatic labeling tasks for pitaya, eggplant, and cucumber datasets in the following two experimental parts:

(i) Based on Guided-GAN, a fruit image translation network, spanning fruit image translation with large differences in phenotypic features was achieved. Foreground images of large batches of pitaya, eggplant, and cucumber were obtained using pear image translation. The three parts of the experiments are denoted as $\mathrm{pear}2\mathrm{pitaya}$, $\mathrm{pear}2\mathrm{eggplant}$, and $\mathrm{pear}2\mathrm{cucumber}$ (described in detail in the Fruit image translation results section).
(ii) The synthetic dataset is constructed using the simulated fruit foreground images of the target domain generated by the translation. It is subsequently input to the anchor-free target detection algorithm CenterNet [42]. The detection network is updated cyclically using a pseudo-label self-learning method with adaptive threshold selection to optimize the network model. The obtained training weights of the network model can be expressed as ${\mathrm{M}}_{\mathrm{pitaya}}$, ${\mathrm{M}}_{\mathrm{eggplant}}$, and ${\mathrm{M}}_{\mathrm{cucumber}}$, and finally the label information of the actual scene dataset in the target domain is output (described in detail in the Automatic fruit labeling results section).

#### Fruit image translation results

The translation results of fruit images will directly determine the accuracy of the automatic labeling task. The main goal of this study is to achieve spanning translation between fruit phenotypic features, so we verify the effectiveness of the proposed method by using duck pear as the source domain and performing the translation of pitaya, eggplant, and cucumber images to achieve the translation between fruits with large feature differences. We also compare with the fruit translation models CycleGAN and Across-CycleGAN, which performed well in the previous studies EasyDAM_V1 and EasyDAM_V2.

**Figure 4:**
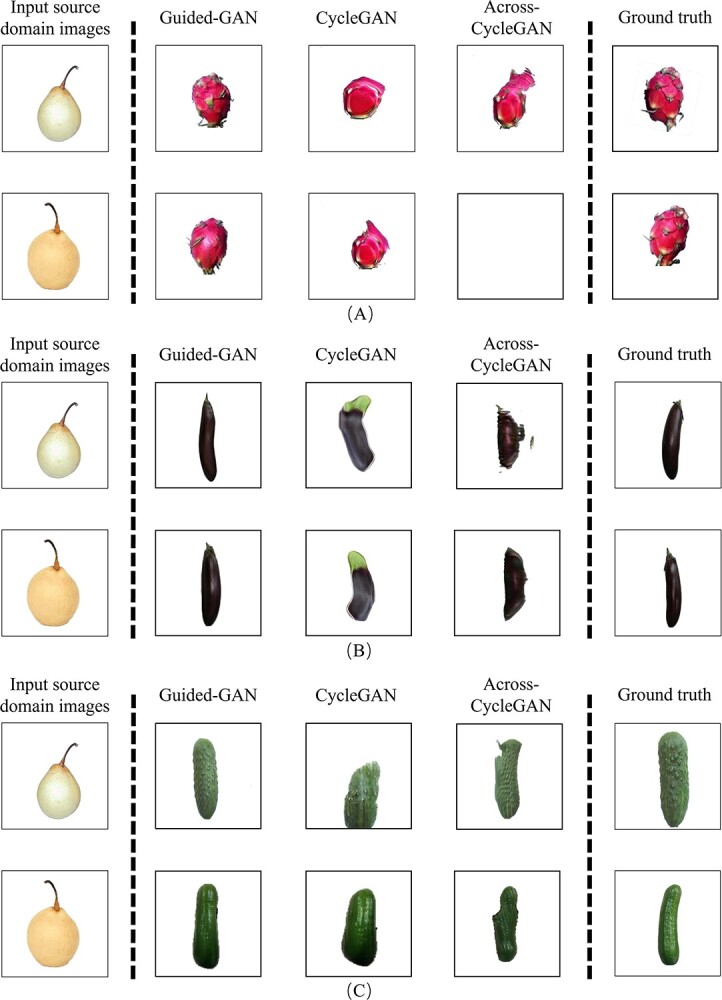
Visualization results of fruit translation performed by different models. **A** is the translation result of the pear2pitaya experiment, **B** is the translation result of the pear2eggplant experiment, and **C** is the translation result of the pear2cucumber experiment.

In this paper, we used 561 pear images with transparent background to train three models, Guided-GAN, CycleGAN, and Across-CycleGAN. To better match the target domain dataset, we conducted two pear2cucumber experiments. The images of cucumber with and without white tubercle spines were generated by translation. Figure 4 shows the visualization results of translating pear images to pitaya, eggplant, and cucumber images using different models in this study in turn. It can be seen that there are extremely obvious differences between the source domain fruit images and the real target domain fruit images in terms of features such as shape, texture, and color. This is a great challenge for the image translation model.

For the pear2pitaya experiment, the pitaya sample exhibits extremely complex texture and shape features in the 2D image due to the presence of a large number of scale structures. This is a major challenge for the GAN to accurately characterize the fruit features. As can be seen in Fig. 4A, the CycleGAN model generates images that are more realistic in terms of color. However, the shape information of the original image is retained, resulting in less than ideal translation results. In contrast, Across-CycleGAN adds a structural similarity loss function across cycles. It shows a large difference in shape compared with the source domain image. But there is also the problem of image distortion.

For the pear2eggplant experiment, the texture features of the eggplant image are not complex; however, the color and shape features differ significantly from the pear image in the source domain. It can be seen in Fig. 4B that the CycleGAN model, which performs well in coloring, has distortions in color. This is mainly due to the fact that the CycleGAN model is not sufficient to support it for feature translation across a large span of time. This ultimately leads to unsatisfactory results. The Across-CycleGAN model, on the other hand, ensures that the color features are successfully transformed with a large variation in shape. But the translated results have some noise interference. This is mainly because the problem of weight leveling of different loss functions can have some impact on the generated images. This is not ideal for unsupervised automatic labeling tasks.

**Figure 5:**
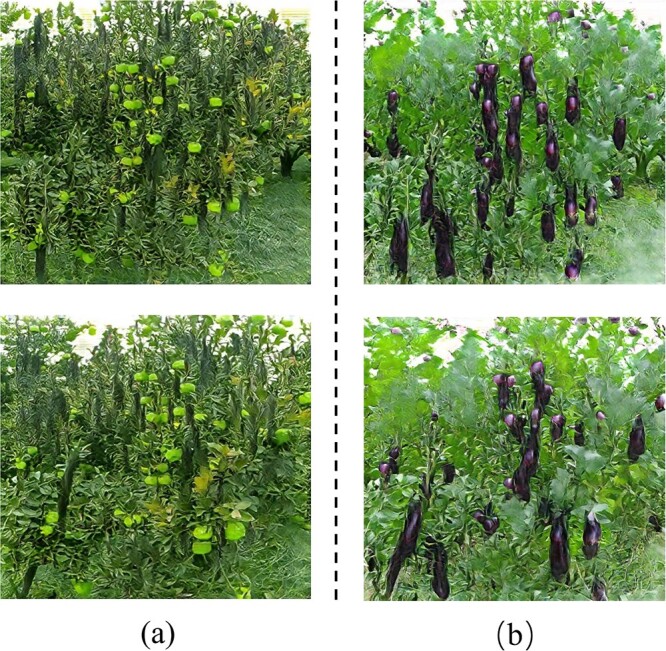
Target domain synthesized dataset images by EasyDAM_V2. **A** Cucumber synthesized image. **B** Eggplant synthesized image.

For the pear2cucumber experiment, this study conducted translation experiments for thorny cucumber and thornless cucumber. As can be seen from Fig. 4C, the cucumber images generated by both the baseline method CycleGAN and Across-CycleGAN have different degrees of distortion. Although Across-CycleGAN enables large changes in shape features, the constraints of the model do not guarantee that it generates realistic images in the spanning fruit image translation task.

In addition, the number of our proposed Guided-GAN parameters is 12.83 MB with 119.83 GFLOPS, and 4 GB of RAM. Meanwhile, the number of Across-CycleGAN parameters is 13 MB with 124.62 GFLOPS, and 4GB of RAM. The number of CycleGAN parameters is 11.38 MB with 116.45 GFLOPS, and 4GB of RAM. The number of Guided-GAN parameters is slightly larger than that of the CycleGAN used in EasyDAM_V1. With a slightly smaller number of parameters than the Across-CycleGAN used in EasyDAM_V2, it achieves better experimental transformation results.

In summary, it can be seen that we cannot achieve satisfactory results using other baseline schemes in the spanning fruit translation task with large differences in phenotypic features. In contrast, the Guided-GAN model proposed in this paper can accurately characterize the phenotypic features of fruits during the training process by introducing a feature map of shape textures and a multi-dimensional feature loss function. Constraints are placed on the process of generating images, thus achieving results in multi-class fruit translation with large differences in phenotypic features.

#### Automatic fruit labeling results

In this section of experiments, we implement an unsupervised automatic labeling task for pitaya, eggplant, and cucumber datasets. We construct synthetic datasets using the target domain simulated fruit foreground images generated by the translation. Subsequently, they were input to CenterNet [42], an anchor-free-based target detection algorithm. The detection network is updated cyclically using a pseudo-label self-learning method with adaptive threshold selection to optimize the network model. The obtained training weights of the network model can be denoted as ${\mathrm{M}}_{\mathrm{pitaya}}$, ${\mathrm{M}}_{\mathrm{eggplant}}$, and ${\mathrm{M}}_{\mathrm{cucumber}}$. The final output is the label information of the actual scene dataset in the target domain.

We then input the synthetic images and their label information into the CenterNet detection model for pre-training. The label frame information is optimized using a pseudo-label self-learning method with adaptive threshold selection strategy. The trained models ${\mathrm{M}}_{\mathrm{pitaya}},{\mathrm{M}}_{\mathrm{eggplant}},\mathrm{and}\ {\mathrm{M}}_{\mathrm{cucumber}}$ are input to the actual orchard images for testing detection accuracy. The visualization results of label generation in this study are shown in Fig. 7.

**Figure 6:**
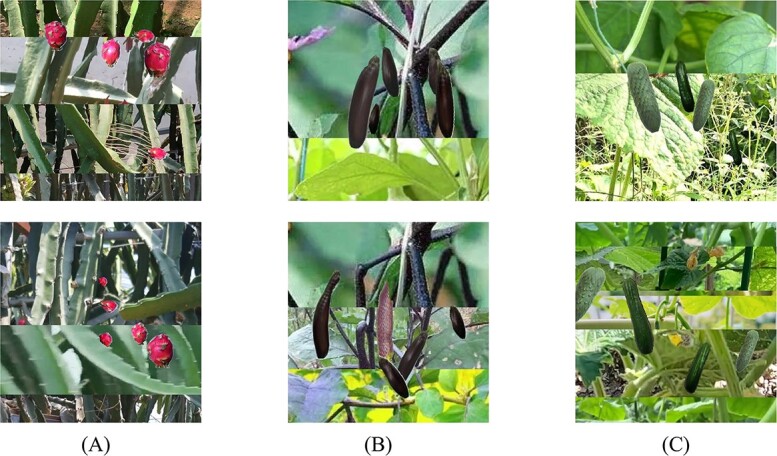
Synthetic dataset images in the target domain. **A** is the synthetic image of pitaya, **B** is the synthetic image of eggplant, and **C** is the synthetic image of cucumber.

**Figure 7:**
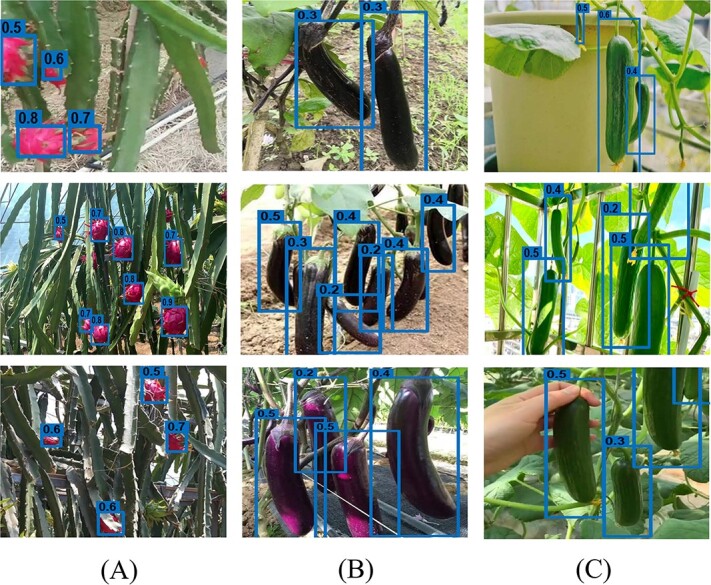
Visualization images of pitaya, eggplant, and cucumber label generation effects under different shooting angles and environments. The number of labeled boxes in the figure is the confidence of that labeled box. **A** Image of pitaya scene. **B** Image of eggplant scene. **C** Image of cucumber scene.

For the ${\mathrm{M}}_{\mathrm{pitaya}}$ experiment, the automatic labeling EasyDAM series method from the previous study was used as the baseline method for comparison in this study. For the eggplant and cucumber datasets, due to the large differences in phenotypic features between the source and target domain, it is more difficult for the method in the previous study EasyDAM_V2 [8] to generate complete fruit images in large batches. As shown in Fig. 5, the converted cucumber and eggplant images differ greatly from the real images and lack fidelity, which makes it difficult to carry out subsequent experiments. Therefore, the EasyDAM series methods are not used for comparison in this study. Figure 6 shows the synthetic dataset images in the target domain. Figure 7 shows the visualization results of our automatic labeling in the three datasets. As can be seen from the figure, for the pitaya labeled boxes, the confidence is mostly above 0.5, indicating that the algorithm has a high degree of certainty about the labeling results. In contrast, the confidence for the cucumber and eggplant labeled boxes is mostly below 0.5, indicating that the algorithm lacks a high degree of certainty about the labeling results for these targets. So pitaya has a higher labeling accuracy.

Table 2 shows the results of the automatic labeling method proposed in this study compared with the previous research methods. In this table, ‘EasyDAM_V2’ method denotes the baseline method used for the comparison experiments. ‘Synthetic dataset method’ denotes the labeling accuracy obtained by training the detection network using synthetic dataset only. ‘Proposed’ denotes the final label generation accuracy obtained by training with the pseudo-labeling self-learning method after fruit image translation using Guided-GAN.

**Table 2:** Label generation results obtained in the actual scene datasets of the three target domains and compared with the EasyDAM_V2 method. (mAP: Mean Average Precision).

**Experiment**	**Test set**	**Method**	**Precision↑**	**Recall↑**	**F1 score**	**mAP↑**
${\mathrm{M}}_{\mathrm{pitaya}}$	Actual orchard pitaya images	EasyDAM_V2 method	0.782	0.778	0.780	0.821
		Synthetic dataset method	0.682	0.682	0.682	0.628
		**Proposed**	**0.871**	**0.868**	**0.869**	**0.878**
${\mathrm{M}}_{\mathrm{eggplant}}$	Actual orchard eggplant images	Synthetic dataset method	0.711	0.719	0.715	0.6912
		**Proposed**	**0.834**	**0.838**	**0.836**	**0.870**
${\mathrm{M}}_{\mathrm{cucumber}}$	Actual orchard cucumber images	Synthetic dataset method	0.637	0.637	0.637	0.648
		**Proposed**	**0.738**	**0.753**	**0.777**	**0.807**

We have analyzed and discussed the following results. In three different experiments, we achieved better results in four metrics—precision, recall, F1 value, and average precision—using the method proposed in this study. The average precision in the ${\mathrm{M}}_{\mathrm{pitaya}}$ experiment is 0.878, which is a 5.7% improvement compared with the baseline method. This is a 25% improvement compared with the synthetic dataset-only method. The average precision in the ${\mathrm{M}}_{\mathrm{eggplant}}$ experiment is 0.870, which is a 17.88% improvement over the synthetic dataset-only method. The average precision in the ${\mathrm{M}}_{\mathrm{cucumber}}$ experiment was 0.807, a 15.9% improvement compared with the synthetic dataset-only method. Because the phenotypic characteristics of eggplant and cucumber differed significantly from those of pear fruit images, the pre-baseline method EasyDAM_V2 was unable to achieve the desired results, so it was not compared with them in these two parts of the experiments.

In addition, it can be seen that in the ${\mathrm{M}}_{\mathrm{pitaya}}$ experiment the improvement of label generation accuracy is more obvious after using the pseudo-label self-learning method. This is mainly due to the presence of a large number of prominent scales in the pitaya samples. The detection model would predict the scales as separate fruit samples, leading to a decrease in detection accuracy. This part of the noisy labels can be effectively filtered out by the pseudo-label self-learning method. Thus, a higher detection accuracy is achieved. A significant performance improvement is achieved.

In addition, the number of CenterNet parameters is 32.66 MB with 70.12 GFLOPS, and 4 GB of RAM. The confidence is 0.1 when visualizing the analysis.

In summary, the automatic labeling task of fruits with large differences in phenotypic characteristics across target domains can be achieved very effectively using the method proposed in this study. Excellent performance was achieved in pitaya, eggplant, and cucumber datasets. The method can also help agricultural researchers to achieve realistic fruit image translation when the fruit feature distribution is too different to select the most suitable source domain dataset for fruit translation tasks. The method can be better applied to downstream tasks.

## Discussion and conclusions

As summarized in Table 3, we proposed the EasyDAM series, aiming for a zero-cost fruit automatic labeling task. EasyDAM_V3 [9] analyzes the fruit phenotype by quantitatively analyzing multiple dimensions, including shape, texture, and color. Then it compares the variability between different fruits to determine the most suitable source domain for target domain transformation. Thus, the model transformation effect is improved. In this paper a new fruit image transformation model, Guided-GAN, is proposed based on EasyDAM_V3 [9], which transforms more significant phenotypic differences, especially in terms of shape. When the most suitable source domain fruit cannot be selected, realistic fruit images can also be obtained in the target domain, thus further improving fruit label generation. A new fruit image translation model, Guided-GAN, is mainly designed to be able to obtain realistic fruit images in the target domain even when the most suitable source domain fruit cannot be selected for image translation. It is also possible to obtain realistic fruit images in the target domain. The method can correspond to a wider range of target domain fruit classes in automatic labeling tasks. The difficulty of source domain fruit selection is greatly reduced. It has better performance for all fruits with large feature differences. We also compare it with the EasyDAM series method in the previous study and demonstrate the effectiveness of the proposed method in this study.

**Table 3:** Summary of the study.

**Method**	**Type of fruit**	**Algorithm characteristics**
EasyDAM_V1 [7]	Source domain: orange Target domain: apple, tomato	Based on the image transformation model CycleGAN, it achieves high-precision automatic annotation of fruit scenes with similar fruit shapes in the source domain and fruit in the target domain.
EasyDAM_V2 [8]	Source domain: orange Target domain: mango, pitaya	Design the Across-CycleGAN. Through the MSSSIM structural consistency loss function and the cross-cycle loss function comparison path, it achieves high-precision automatic annotation of fruit scenes with miniature deformation of fruit in the source domain and fruit in the target domain.
EasyDAM_V3 [9]	Source domain: pear Target domain: orange, apple, tomato	Propose the concept of optimal source domains for the first time. The most suitable source domain fruit dataset is selected for the target domain using a multidimensional spatial feature model. And propose a large-volume dataset construction method, which can obtain fruit labeling information automatically.
EasyDAM_V4	Source domain: pear Target domain: pitaya, cucumber, eggplant	Design the Guided-GAN. Through the multi-dimensional phenotype feature extraction method based on latent space and multi-dimensional loss function design method based on the entropy weight method, it achieves high-precision automatic annotation of fruit scenes with large deformation of fruit in the source domain and fruit in the target domain.

To validate the effectiveness of the method in this study for the task of automatic labeling of spanning fruits with large differences in phenotypic features, we chose to use the pear dataset as the source domain and the pitaya, eggplant, and cucumber datasets as the target domains. The Guided-GAN model was used for fruit image translation, and we compared it with the CycleGAN and Across-CycleGAN models. It can be seen from Fig. 7 and Fig. S1–2 in Supplementary Data that the Guided-GAN model achieves the best results. The other methods cannot achieve satisfactory results in the fruit translation task with large feature differences. Therefore, the Guided-GAN fruit image translation model proposed in this paper can achieve realistic image translation with large differences in features, especially in shape features. Subsequently, in order to demonstrate the effectiveness of the automatic labeling method more intuitively, we use a synthetic dataset construction method and a pseudo-label self-learning method for automatic labeling of the target domain dataset for the translated images. Average precision of 87.8% was achieved in the pitaya dataset using the method of this study, which is a 5.7% improvement compared with the previous study method. Average accuracy of 87.0% was achieved in the eggplant dataset. In the cucumber dataset, the average precision of 80.7% was achieved. It has certain value and significance in practical production life. In summary, it can be seen that the Guided-GAN fruit translation model proposed in this study can achieve more realistic leapfrog fruit target translation with large differences in phenotypic characteristics while ensuring the diversity of generated samples. It provides more realistic and effective target foreground features for the subsequent fruit detection models. Thus, the labeling accuracy of the automatic fruit labeling task is improved. In addition, since this study can achieve large shape translation, it significantly improves the applicability of the automatic labeling method and reduces the difficulty of fruit selection in the source domain. The EasyDAM_V4 method is also able to perform the automatic labeling task accurately and efficiently when the best source domain dataset cannot be selected due to the discrete feature distribution.

Meanwhile, from the results, we found that the automatic labeling accuracy of eggplant and cucumber is lower compared with pitaya. This is due to the fact that both eggplant and cucumber targets are long. The detection frame is relatively large in length and width. The color characteristics of the cucumber target are very similar to the background. All these factors make it more difficult to train the detection network. The detection accuracy is slightly reduced compared with pitaya samples. When applying our method, there are several points to consider. During the image acquisition and processing stage, incorrect operation in photographing should be avoided. Make the fruit as clear as possible in the image and reduce blurring caused by camera movement. During the image selection stage, try to select the fruit images with less occlusion to avoid serious mutilation or deformation of the fruit. In recent studies, we have noticed that some studies have used anchorless frame detectors and balanced positive and negative samples to improve the detection performance of fruit targets with relatively large aspect [43, 44]. We will subsequently study and discuss this research idea to improve the fruit detection model and improve the performance of automatic labeling methods.

Several substantial challenges persist in practical orchard applications, such as yield estimation and fruit harvesting, including the complexities associated with implementing deep learning algorithms and the protracted duration required for fruit phenotypic parameter analysis [45, 46]. Notably, within these challenges, the extensive training times demanded by deep learning models and the prohibitive expenses linked to manual labeling emerge as pivotal determinants [47, 48]. The automatic labeling method introduced in this study demonstrates adaptability to diverse orchards and variable environmental conditions. It effectively streamlines the construction of deep learning models, significantly mitigating the burdensome time investments typically associated with training. Furthermore, it circumvents the exorbitant costs linked to manual labeling procedures. Consequently, this method expedites the observation and analysis of essential fruit attributes such as shape and color, thus furnishing a robust foundation of data and innovative technical support for fruit phenotypic analysis.

To promote the widespread use and implementation of our method, we have made the dataset, as well as the model weights, and the test code publicly available in Github. The download link is available. Meanwhile, we plan to develop an online automatic labeling platform to provide other researchers with convenient, efficient, and accurate automatic labeling tools in the future. The platform will enable researchers to upload fruit images to be labeled on demand and with guided manipulation. Thus, the task of automatic labeling of different fruit images is accomplished.

## Acknowledgements

This study was partially supported by the National Natural Science Foundation of China (NSFC) Program 62276009 and the Japan Science and Technology Agency (JST) AIP Acceleration Research JPMJCR21U3.

## Author contributions

W.Z., Y.L., and W.G. conceived the ideas and designed the methodology; W.Z., Y.L., and W.G. implemented the technical pipeline, conducted the experiments, and analyzed the results; Y.L. and C.W. analyzed the data with input from W.Z. and W.G.; C.W., C.Z., and G.C. conducted the supplementary experiment. All authors discussed and wrote the manuscript and provided final approval for publication.

## Data availability

The dataset and pretrained model used in this paper are available at https://github.com/I3-Laboratory/EasyDAM_dataset.

## Conflict of interest

The authors declare that they have no conflicts of interest.

## Supplementary data

Supplementary data is available at *Horticulture Research* online.

## Supplementary Material

Web_Material_uhae007

## References

[1] Boquete MT , Muyle A, Alonso C. Plant epigenetics: phenotypic and functional diversity beyond the DNA sequence. *Am J Bot*. 2021;108:553–8 33887061 10.1002/ajb2.1645

[2] Muthukumar P , Jat GS, Kalia P. et al. Morphological characterization and screening of *Solanum habrochaites* accessions for late blight (*Phytophthora infestans*) disease resistance. *Genet Resour Crop Evol*. 2023;1–9

[3] Munaweera TIK , Jayawardana NU, Rajaratnam R. et al. Modern plant biotechnology as a strategy in addressing climate change and attaining food security. *Agric Food Sec*. 2022;11:1–28

[4] Sun G , Lu H, Zhao Y. et al. AirMeasurer: open-source software to quantify static and dynamic traits derived from multiseason aerial phenotyping to empower genetic mapping studies in rice. *New Phytol*. 2022;236:1584–604 35901246 10.1111/nph.18314 PMC9796158

[5] Zhu Y , Sun G, Ding G. et al. Large-scale field phenotyping using backpack LiDAR and CropQuant-3D to measure structural variation in wheat. *Plant Physiol*. 2021;187:716–38 34608970 10.1093/plphys/kiab324 PMC8491082

[6] Zahir SADM , Omar AF, Jamlos MF. et al. A review of visible and near-infrared (Vis-NIR) spectroscopy application in plant stress detection. *Sensors Actuators A Phys*. 2022;338:113468

[7] Zhang W , Chen K, Wang J. et al. Easy domain adaptation method for filling the species gap in deep learning-based fruit detection. *Hortic Res*. 2021;8:119 34059636 10.1038/s41438-021-00553-8 PMC8167097

[8] Zhang W , Chen K, Zheng C. et al. EasyDAM_V2: efficient data labeling method for multishape, cross-species fruit detection. *Plant Phenomics*. 2022;2022:9761674 36204392 10.34133/2022/9761674 PMC9513831

[9] Zhang W , Liu Y, Zheng C. et al. EasyDAM_V3: automatic fruit labeling based on optimal source domain selection and data synthesis via a knowledge graph. *Plant Phenomics*. 2023;5:0067 37519937 10.34133/plantphenomics.0067 PMC10374194

[10] Shamsolmoali P , Zareapoor M, Granger E. et al. Image synthesis with adversarial networks: a comprehensive survey and case studies. *Inf Fusion*. 2021;72:126–46

[11] Mo S , Cho M, Shin J. InstaGAN: Instance-aware image-to-image translation. Proc Int Conf Learn Represent (ICLR). 2018:1–70

[12] Chen Y , Xia S, Zhao J. et al. Appearance and shape based image synthesis by conditional variational generative adversarial network. *Knowl Based Syst*. 2020;193:105450

[13] Roy P , Häni N, Isler V. Semantics-aware image to image translation and domain transfer. arXiv preprint arXiv:1904.02203. 2019

[14] Chen Z , Kim VG, Fisher M. et al. DECOR-GAN: 3D shape detailization by conditional refinement. In: Proceedings of the 2021 IEEE/CVF Conference on Computer Vision and Pattern Recognition. 2021, 15735–44

[15] Wu R . Geometry-aware image-to-image translation. Dissertation,. The Chinese University of Hong Kong; 2021:

[16] Li R , Li X, Hui K-H. et al. SP-GAN: sphere-guided 3D shape generation and manipulation. *ACM Trans Graphics*. 2021;40:151

[17] Zhang J , Hou J. Unpaired image-to-image translation network for semantic-based face adversarial examples generation. In: Proceedings of the 2021 Symposium on Great Lakes Symposium on VLSI. 2021, 449–54

[18] Gokaslan A , Ramanujan V, Ritchie D. et al. Improving shape deformation in unsupervised image-to-image translation. In: *Proceedings of the European Conference on Computer Vision (ECCV)*. 2018:649–65

[19] Huang S , He C, Cheng R. SoloGAN: multi-domain multimodal unpaired image-to-image translation via a single generative adversarial network. *IEEE Trans Artif Intell*. 2022;3:722–37

[20] Hedjazi MA , Genc Y. Efficient texture-aware multi-GAN for image inpainting. *Knowl Based Syst*. 2021;217:106789

[21] Hu X . Multi-texture GAN: exploring the multi-scale texture translation for brain MR images. arXiv preprint arXiv:2102.07225. 2021

[22] Karras T , Laine S, Aila T. A style-based generator architecture for generative adversarial networks. In: Proceedings of the IEEE/CVF Conference on Computer Vision and Pattern Recognition. 2019:4401–10 10.1109/TPAMI.2020.2970919 32012000

[23] Karras T , Laine S, Aittala M. et al. Analyzing and improving the image quality of styleGAN. In: Proceedings of the IEEE/CVF Conference on Computer Vision and Pattern Recognition. 2020:8110–9

[24] Karras T , Aittala M, Laine S. et al. Alias-free generative adversarial networks. *Adv Neural Inf Proces Syst*. 2021;34:852–63

[25] Wu X , Shao J, Gao L. et al. Unpaired image-to-image translation from shared deep space. In: 25th IEEE International Conference on Image Processing (ICIP). 2018:2127–31

[26] Johnson J , Alahi A, Fei-Fei L. Perceptual losses for real-time style transfer and super-resolution. In: Computer Vision–ECCV 2016: 14th European Conference, Amsterdam, The Netherlands, October 11-14, 2016, Proceedings, Part II 14. Springer: Cham, 2016,694–711

[27] Liu M-Y , Breuel T, Kautz J. Unsupervised image-to-image translation networks. Proc 31st Int Conf Neural Inf Process Syst. 2017:700–8

[28] Bergmann U , Jetchev N, Vollgraf R. Learning texture manifolds with the periodic spatial Gan. Proc Int Conf Mach Learn. 2017:469–77

[29] Shen Y , Yang C, Tang X. et al. InterFaceGAN: interpreting the disentangled face representation learned by GANs. *IEEE Trans Pattern Anal Mach Intell*. 2020;44:2004–18 10.1109/TPAMI.2020.3034267 33108282

[30] Sainburg T , Thielk M, Theilman B. et al.. Generative adversarial interpolative autoencoding: Adversarial training on latent space interpolations encourage convex latent distributions. arXiv:1807.06650. 2018

[31] Chen X , Duan Y, Houthooft R. et al. InfoGAN: interpretable representation learning by information maximizing generative adversarial nets. *Adv Neural Inf Proces Syst*. 2016;29,https://arxiv.org/abs/1606.03657

[32] Bengio Y , Courville A, Vincent P. Representation learning: a review and new perspectives. *IEEE Trans Pattern Anal Mach Intell*. 2013;35:1798–828 23787338 10.1109/TPAMI.2013.50

[33] Selvaraju RR , Cogswell M, Das A. et al. Grad-cam: visual explanations from deep networks via gradient-based localization. In: Proceedings of the IEEE International Conference on Computer Vision. 2017:618–26.

[34] Sosa J , Buitrago L. A review of latent space models for social networks. *Rev Colomb Estad*. 2021;44:171–200

[35] Kim B , Lee KH, Xue L. et al. A review of dynamic network models with latent variables. *Stat Surv*. 2018;12:105 31428219 10.1214/18-SS121 PMC6699782

[36] Bojanowski P , Joulin A, Lopez-Paz D. et al.. Optimizing the latent space of generative networks. Proc Int Conf Mach Learn. 2018:600–9

[37] Ma S , Fu J, Chen CW. et al. DA-GAN: instance-level image translation by deep attention generative adversarial networks. In: Proceedings of the IEEE Conference on Computer Vision and Pattern Recognition. 2018:5657–66

[38] Shen Z , Huang M, Shi J. et al. Towards instance-level image-to-image translation. In: Proceedings of the IEEE/CVF Conference on Computer Vision and Pattern Recognition. 2019:3683–92

[39] Wang Z , Simoncelli EP, Bovik AC. Multiscale structural similarity for image quality assessment. In: The Thirty-Seventh IEEE Asilomar Conference on Signals, Systems and Computers. 2003;2:1398–402

[40] Thompson A. Fruits-360 dataset *.* https://www.kaggle.com/moltean/fruits, 2017.(18 May 2020, date last accessed)

[41] Minut M-D . Fruits-262 dataset: a dataset containing a vast majority of the popular and known fruits*.* https://www.kaggle.com/datasets/f9472b258bbdab0dbc8cc773ad8c78a2fa1b997fa0cd88a476f184b78b93338c, 2021. (25 May 2021, date last accessed)

[42] Zhou X , Wang D, Krähenbühl P. Objects as points. arXiv 2019

[43] Tian Z , Shen C, Chen H. et al. FCOS: fully convolutional one-stage object detection. In: Proceedings of the IEEE/CVF International Conference on Computer Vision. 2019:9627–36

[44] Ge Z , Liu S, Wang F. et al.. YOLOX: exceeding YOLO series in 2021. arXiv preprint arXiv:2107.08430 2021

[45] Singh B , Dhinakaran DP, Vijai C. et al. Artificial intelligence in agriculture. *J Surv Fish Sci*. 2023;10:6601–11

[46] Hu F , Lin C, Peng J. et al. Rapeseed leaf estimation methods at field scale by using terrestrial LiDAR point cloud. *Agronomy*. 2022;12:2409

[47] Meshram V , Patil K, Meshram V. et al. Machine learning in agriculture domain: a state-of-art survey. *Artif Intell*. *Life Sci*. 2021;1:100010

[48] Montoya-Cavero L-E , de León D, Torres R. et al. Vision systems for harvesting robots: produce detection and localization. *Comput Electron Agric*. 2022;192:106562

