# Supplementary material for: EasyDAM_V4: Guided-GAN-based cross-species data labeling for fruit detection with significant shape difference
Source: Hortic Res. 2024 Jan 10;11(3):uhae007. doi: 10.1093/hr/uhae007 (PMC10939356; doi:10.1093/hr/uhae007)
Supplement: Web_Material_uhae007 [file web_material_uhae007.zip › Data description of EasyDAM_V4_20131019.docx]

# Data description

## Description of the fruit image dataset used in EasyDAM_V4

This folder contains the dataset used in the paper 《EasyDAM_V4: Guided-GAN based cross-species data labeling for fruit detection with significant shape difference》, which mainly includes the fruit image dataset used to train the GAN network and the fruit image dataset used for object detection.

### GAN dataset

- **Data content:**

This fruit dataset is used to train the generative adversarial network Guided-GAN to achieve the source domain fruit to the target domain fruits. Including pear2pitaya, pear2eggplant and pear2cucumber. Each dataset contains 8 image files for GAN network training：trainA， trainA_gray， trainB, trainB_gray, testA, testA_gray, testB, and testB_gray. A fruit image containing only the foreground was used as the dataset. The resolution in the dataset is uniformly 256*256 to allow for feature analysis and deep learning model training. The images in the dataset are obtained from the public dataset fruit360, actual fruit collections, and Internet searches (no copyright restrictions).

- **Data collection location:** Internet, Beijing University of Technology, Beijing, China.
- **Data Collection Objects:** pear, cucumber, eggplant, pitaya.
- **Data collection methods:** Internet, cell phone photography.
- **Data collection device:** cell phone.
- **Data format:** jpg, png.

### Object detection dataset

Fruit images of different scenes taken in real orchards with fruit species, including cucumber, eggplant, and pitaya, are used as a dataset for training and testing the fruit detection model. Among them, each fruit dataset contains two types of synthetic dataset (used to train the detection model) and real scene dataset (used to validate the model). And each synthetic dataset has 1000 images.

- **Pitaya dataset**
- **Data content:**

The dataset contains 377 pitaya images. Among them, 265 unlabeled images are used as the training set for the pseudo-labeling self-learning method, and 112 labeled images are used as the test set to verify the effectiveness of the proposed automatic labeling method.

- **Data collection location:** Internet, Shengpu Cheng Sightseeing and Picking Garden, Gu Zheng Road, Beijing, China.
- **Data collection methods:** Internet, cell phone photography.
- **Data collection device:** cell phone.
- **Data format:** jpg, png.
- **Eggplant dataset**
- **Data content:**

This dataset contains 516 eggplant images of different varieties from different angles. Among them, 380 unlabeled images are used as the training set for the pseudo-label self-learning method, and 136 labeled images are used as the test set.

- **Data collection methods:** Fruit-262 public dataset, Internet.
- **Data format:** jpg, png.
- **Cucumber dataset**
- **Data content:**

There are 380 images of cucumbers with white spines and without spines. Among them, 297 unlabeled images were used as the training set for the pseudo-labeling self-learning method, and 83 labeled images were used as the test set.

- **Data collection location:** Internet, Shengpu Cheng Sightseeing and Picking Garden, Gu Zheng Road, Beijing, China.
- **Data collection methods:** Internet, cell phone photography.
- **Data collection device:** cell phone.
- **Data format:** jpg, png.
